# Ground penetrating radar: a case study for estimating root bulking rate in cassava (*Manihot esculenta* Crantz)

**DOI:** 10.1186/s13007-017-0216-0

**Published:** 2017-08-07

**Authors:** Alfredo Delgado, Dirk B. Hays, Richard K. Bruton, Hernán Ceballos, Alexandre Novo, Enrico Boi, Michael Gomez Selvaraj

**Affiliations:** 1 Department of Soil and Crop Sciences, Texas A&M University, College Station, TX USA; 2 International Center for Tropical Agriculture (CIAT), Cali, Colombia; 3 IDS GeoRadar North America, Golden, CO USA

**Keywords:** Ground penetrating radar, Cassava, Roots, High throughput phenotyping, Bulking rate

## Abstract

**Background:**

Understanding root traits is a necessary research front for selection of favorable genotypes or cultivation practices. Root and tuber crops having most of their economic potential stored below ground are favorable candidates for such studies. The ability to image and quantify subsurface root structure would allow breeders to classify root traits for rapid selection and allow agronomist the ability to derive effective cultivation practices. In spite of the huge role of Cassava (*Manihot esculenta* Crantz), for food security and industrial uses, little progress has been made in understanding the onset and rate of the root-bulking process and the factors that influence it. The objective of this research was to determine the capability of ground penetrating radar (GPR) to predict root-bulking rates through the detection of total root biomass during its growth cycle. Our research provides the first application of GPR for detecting below ground biomass in cassava.

**Results:**

Through an empirical study, linear regressions were derived to model cassava bulking rates. The linear equations derived suggest that GPR is a suitable measure of root biomass (*r* = .79). The regression analysis developed accounts for 63% of the variability in cassava biomass below ground. When modeling is performed at the variety level, it is evident that the variety models for SM 1219-9 and TMS 60444 outperform the HMC-1 variety model (r^2^ = .77, .63 and .51 respectively).

**Conclusions:**

Using current modeling methods, it is possible to predict below ground biomass and estimate root bulking rates for selection of early root bulking in cassava. Results of this approach suggested that the general model was over predicting at early growth stages but became more precise in later root development.

## Background

Cassava (*Manihot esculenta* Crantz) is a tropical root crop originally from South America [1] that serves as a staple food source for an estimated 800 million people [2]. More than a tenth of the world’s population relies on this food source, and in tropical countries, it follows only maize and rice in caloric intake [3]. Worldwide, cassava is the second most important source of starch after maize [4]. Between 1991–1993 and 2011–2013, the global harvested area of cassava expanded by 25%, from 16.5 million to 20.7 million hectares, which was the biggest percentage increase among the world’s five major food crops. Most of this cultivated increase occurred in Africa (with an increase of 39.2%), which alone produces nearly 145 million metric tons of cassava per year. It is now considered the fourth most important food crop and an essential dietary component of millions across the world [5].

South East Asia’s (particularly Thailand, Cambodia, and Vietnam) average fresh root yields have almost doubled in the last 20 years: average yield in 1994 was 12.01 t ha^−1^ whereas in 2014 root productivity was 21.5 t ha^−1^ [6]. In spite of the yield potential of new varieties, average productivity across Sub-Saharan Africa has increased only marginally between the 1994 and 2014 period from 8.1 to 8.4 t ha^−1^ [6]. Several reasons may explain the greater gains in productivity observed in SE Asia. There are no major biotic stresses for cassava in that region compared with Africa, where diseases and pests limit productivity drastically. In Africa, cassava is a key food security crop, often grown in association with few other species, and cooking quality may have a higher priority than yield in farmers’ preference. Breeders in Africa, therefore have to compromise increases in yield with quality traits. In SE Asia, on the other hand, cassava is mostly an industrial crop used for the production of starch and dried chips which allow the breeders to concentrate basically on high fresh root yield, high dry matter content and adequate plant architecture [4, 7]. Strong markets in SE Asia encourage the adoption of new technologies (e.g. highly productive varieties and appropriate cultivation practices). A large proportion of the area planted to cassava in SE Asia, therefore, is with improved varieties. In Africa, adoption of improved varieties has been limited by the understandable and common reluctance of farmers to change practices for which their food security depends on. Taken together, it is evident that the potential for higher income could be significant if improved varieties are introduced; even in regions of low agriculture inputs [7, 8].

Limited information is available on growth patterns in cassava roots as compared to aboveground biomass [9, 10]. This is a serious constraint considering the root is the main commercial product. A major constraint that cassava breeding programs have is the low multiplication rate for the planting material (stem cuttings). This lack of planting material results in a lengthy evaluation schedule that requires several years until multi-location trials can be conducted [4, 7]. This limitation and the need to perform staged destructive samplings also restrict breeders’ and agronomists’ ability to screen root development and bulking rates through the growing season. As such, cassava researchers are in need of new rapid, non-destructive procedures to capture root phenotypic data [7].

A relevant and descriptive trait that needs to be captured in cassava is the root-bulking rate (RBR). RBR can be defined as the rate of change in mass over time. A non-destructive protocol that captures RBR could facilitate discrimination of high yielding, early bulking varieties that would increase yields and profits, allow for alternative cropping systems (crop associations and rotations) as well as optimizing varietal response to management practices. By monitoring root mass over time for plants undergoing a biotic or abiotic stress, non-destructive methods could also prove useful in breeding for tolerance or resistance.

Most models currently used for the estimation of root bulking rate or various other root growth functions are based on vegetative characteristics that are not highly predictive of root production and vary between varieties due to developmental asynchrony, genotype by environment interactions (G × E), and a lack of knowledge in source-sink relationships that drive root bulking [11]. Root productivity growth models and variety selections are based on an endpoint harvest cycle of 11–12 months after planting and cultivars that have already been selected based on productivity at this stage. This type of selection and model development cannot identify early bulking clones which are often requested by farmers, particularly in Africa. Additionally, current models are poor surrogates for root growth rate determination across all environments and genotypes. Also, any early work through the empirical study of RBR has been dependent on temporal measurements of root mass and destructive harvest [7]. The destructive sampling requires large populations and trials that are laborious, expensive, and preclude, large germplasm screens, or multi-location and large entry trials. The ability to determine RBR across environments in a rapid non-destructive process would reduce trial size requirements, cutting time and cost in phenotypic data capture for selection of early bulking cassava varieties. The objective of this study was to determine the capability of ground penetrating radar (GPR) to image and quantify root mass throughout the root growth cycle as a measure of root-bulking rate.

GPR is an existing and rapidly evolving technology that can be used as a high throughput (HT), non-destructive-3-dimensional imaging method—for quantifying cassava root mass. Most GPR systems work in a time domain function by emitting electromagnetic pulses into the ground in which part of the energy is reflected, transmitted or scattered at boundaries of contrasting materials [12, 13]. The reflected strength of the return is recorded as a function of travel time [13]. Many thousands of measurements are acquired across a planned grid network by moving the antenna along a ground transect at fixed intervals. These returns can be quantified and rendered into a 3-D field allowing for visualization and mapping of belowground root biomass. With the ability to detect subtle differences in the soil media GPR has often been utilized as a small cross-section near-surface object detection tool [14–18]. GPR technology has been utilized to nondestructively image coarse root biomass and architecture previously in various tree and shrub species [19–22]. In adapting this technology for temporal non-destructive sampling, the tool can be utilized as a proxy for RBR detection and facilitate genotype characterization at different growth stages and in responses to novel cultivation, irrigation, and fertilization practices.

## Methods

### Study Site

The study site was located at the International Center for Tropical Agriculture (CIAT) in Palmira, Valle del Cauca, Colombia. The site is more specifically located at 3°29′ North and 76°21′ West at an approximate altitude of 1020 m.a.s.l. Temperatures at the study site range from 19 to 30 °C. The site has bimodal rainfall with peaks occurring between March–June and October–December and an annual average of 1 m. The trials were conducted in an ongoing nursery in which the plantings had been established in two separate fields. The soil of field one is a fertile alluvial clay loam while field two is a sandy clay loam.

### Planting materials

The data was collected as a subset of a larger nursery established by the cassava breeding program at CIAT. The trial was planted at monthly intervals to have a constant availability of flowers. The subset data was collected from four planting dates (December 2013, January 2014, February 2014, and March 2014). The data was collected on May 26, 2014, at which time the plant age for each date would be six, five, four, and three months, respectively. No above ground phenological parameters were collected or utilized in the sampling period selection as the trial was designed to solely capture rate of change in root mass across time. Three varieties of cassava were included in the study, HMC-1, SM 1219-9, and TMS-60444 (hereinafter referred to as HMC, SM, and TMS). HMC, planted in field one, is a commercial variety released in Colombia from a cross first made in 1980. SM, planted in field two, is the result of a poly cross (open pollination) made in 1988 for which only its female progenitor (CG 1450-4), derived from the Colombian landraces MCOL 1505 and MCOL 1940, is known. TMS, planted in field two, originated in Nigeria and has been used as the model genotype for genetic transformation work [23–25]. Selection of these three varieties was based on the observed rooting architecture in which roots were shallow and laterally growing which facilitated capture. Healthy stem cuttings (stakes) ranging from .15 to .20 m long taken from the lower to mid-section of healthy plants were utilized as planting material. Stakes were planted vertically into the soil roughly half their length. Stakes were planted 1 m apart in furrows that were also 1 m apart. This is the common plant spacing utilized in commercial cassava production and was considered beneficial for GPR data processing in that it allowed for ample separation between plants.

### Radar acquisition parameters

Radar acquisition was conducted utilizing an IDS multichannel GPR system (Detector Duo™) which collects information at two frequencies (700 and 250 MHz) with horizontal transmit and horizontal receive polarization (HH Polarization) for both frequencies. The unit has a scan interval of 42 scans/m, a scan rate per channel of 127 s/sec at 512 samples/scan, and a time window of 40 ns. For this trial, only the returns of the 700 MHz frequency antenna were utilized. This frequency theoretically has the most significant returns in regards to resolution across all age cohorts and penetration depth in the field based on its theoretical resolution. The theoretical resolution of the 700 MHz antennae is approximated at .037 m. The calculation for this estimation was derived from Anan [26] in which highest resolution is achieved at one-quarter the wavelength. This is consistent empirically with findings of Cui et al. [27], in which increased frequency provided optimal resolution of roots.

**Fig. 1:**
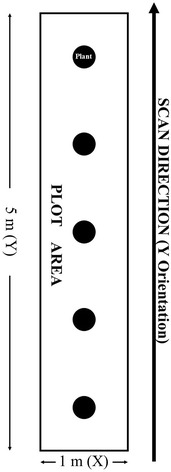
Plot layout in which the *large rectangular area* is considered the extent of the plot (1 m × 5 m). The *five circles* suggest the location of the cassava plants found in the plot, and the *directional arrow* suggests the movement of the ground penetrating radar antenna. Scan line spacing was set at .05 m in the X. The sampling interval was based on a manufacturer’s established setting for which a new pulse is emitted every .013 m in the Y as established by a calibrated measuring wheel attached to the unit

GPR data were collected at a scan line spacing of .05 m in the X or perpendicular to the row direction and a sampling interval of .013 m in the Y direction or parallel to the row (Fig. 1). Scan line spacing was set at .05 m. Though quarter wavelength for 700 MHz frequency assuming an average propagation velocity of .10 m/ns is not met at .05 m spacing, it is the closest and most efficient spacing in regards to accuracy in field movement of the sensor and time in the field. The sampling interval was based on manufacturers established setting for which a new pulse is emitted every .013 m as established by a calibrated measuring wheel attached to the unit. This spacing and sampling interval ensured high-resolution imaging of the plot areas that provide greater detail of below ground structure [28] and the higher frequency antenna (700 MHz) provided necessary vertical resolution for the first 30 cm of soil. This depth would capture a significant extent of the root zone in that the observed maximum bulked root depth across all varieties was an estimated .45 m.

### Field sampling

The staggered planting design allowed for the capture of data that ranged across four planting dates which helped to elucidate the differences of varieties at similar ages as well as bulking change over time within a specific variety. After acquiring the GPR data, plant roots from each plot were hand harvested, root systems were photographed, and weighed individually. This weighing scheme provided root fresh mass (RFM). Estimated root positions for reference to digital images were also obtained at this step by associating the Cartesian position of the plant and root direction, as well as the average depth of the root mass.

Sampled plots were 5 m × 1 m with 5 plants per plot (Fig. 1). This is a reasonable plot size as a selection of clonal evaluation trials in cassava have ranged from 3 to 7 plant plots [4]. This provided a total of 20 plants sampled in field one (HMC) and 40 plants sampled in field two (SM and TMS) for a total of 60 plants sampled. In order to capture the entire root area, the surface needed to be cleared to allow for the antenna system to move freely through the field, current antenna systems including the Detector Duo™, are designed for smooth flat open surfaces and ground coupling to reduce initial backscatter. This meant that all above ground vegetation for each plant was harvested at the soil surface. This is to say that no single plant was recorded across each age cohort. Each cohort had a separate group of five plants sampled. Though this is a destructive sampling procedure, the advancement of new antenna arrays will allow for a non-destructive approach. Our objective was to first determine the capacity for detection for future implementation of high-throughput methods.

### GPR image processing

**Fig. 2:**
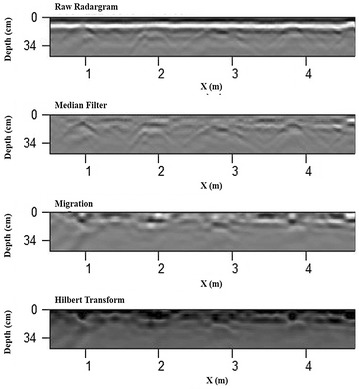
GPR-Slice radargram processing image diagram for 700 MHz antenna returns. From *top* to *bottom*: raw radargram collected in the field, medial filter processed radargram for background noise removal, migrated radargram with collapsed diffractions, and Hilbert transformed radargram illustrated rectified positive domain values

Data processing was performed using the protocol established by Butnor et al. [21]. These procedures are based on digital image processing methodology. The current tool output provides digital images of the time domain function allowing for rapid data processing and visualization of near surface objects. The data processing procedure was performed using GPR-Slice software [29] and MatLab software [30] for filtering procedures, image thresholding, and pixel count. In brief, the method first filtered the raw radargrams for background noise removal using a median background filter (GPR-Slice). Median background filters provided better filtering than standard average scan—background filtering—as the peak responses from cassava reflections would not overweight a median scan used in this subtraction filter. Kirchhoff migrations were then performed on the background filtered data to migrate hyperbolic responses and to collapse diffractions (GPR-Slice). The migrated image was then converted using a Hilbert transform to rectify the pulse data into the pulse envelope (GPR-Slice). The envelope of the pulse defined by the Hilbert transform eliminates the ±nature of the transmit pulse and is used to define regions of just strong or weak reflections as the signal is completely rectified in the positive domain. The transformed data were converted to greyscale images with 256 values, 0 (black) and 255 (white) (MatLab). Known root positions were identified in the images manually by locating the strongest responses that occurred at the same positional distance in an image as defined by the measuring wheel attached to the antenna unit and the actual field-measured distance of a given root using a traditional tape measure from the sensor start position to the position at which a root was harvested. This was done for 50 roots (twenty roots in field one and thirty roots in field two). Based on these estimated root positions, the pixel values in the image associated with a known root location were recorded to derive a mean value for pixel thresholding (MatLab). Utilizing the 95% confidence interval of the mean value (0–85 of the 256 values), thresholding of the greyscale image was performed in which any pixel equal to or less than a value of 85 was given a value of 1, if greater than 85 its was assigned a value of 0. The total count of value 1 pixels was then utilized to determine pixels associated with root presence across all varieties (MatLab). An example showing the 4 main processes applied to raw radargrams and used in the pixel count analysis for this research is given in Fig. 2. For more detail on data processing see JR Butnor, J Doolittle, KH Johnsen, L Samuelson, T Stokes and L Kress [21].

### Statistical analysis

Pearson’s product moment correlation tests were performed for the four input data sets. The first analysis was made using all available data. This provided a general function for determining the correlation between the three cassava varieties and GPR-derived pixel counts. Then individual analyses were made for each variety and the associated RFM. These analyses would test the correlations at a more specific level so as to elucidate any potential issue with the utilization of a general model and to aid in determining if results from a particular variety departed from the general model. Once correlations were derived the regression equations were developed. This procedure aimed at developing linear regression models that best fit the data points. Coefficients of determination were derived and tested for significance by performing a bootstrapping procedure of the data. The coefficient of determination was then found to be significant if the value fell within the 95% confidence interval of the bootstrapped results. To test for prediction accuracy an analysis of variance procedure was performed. This procedure would test if there were significant differences between plant age and RFM of the sampled materials. All statistical analyses were performed in R [31].

A linear regression model approach in which the GPR-derived value (pixel counts) were regressed to RFM to determine correlation and the coefficient of determination to define the root biomass predictive capabilities of GPR. This regression was done utilizing all data sampled for a general predictive model as well as a more specific variety level analysis.

## Results

**Fig. 3:**
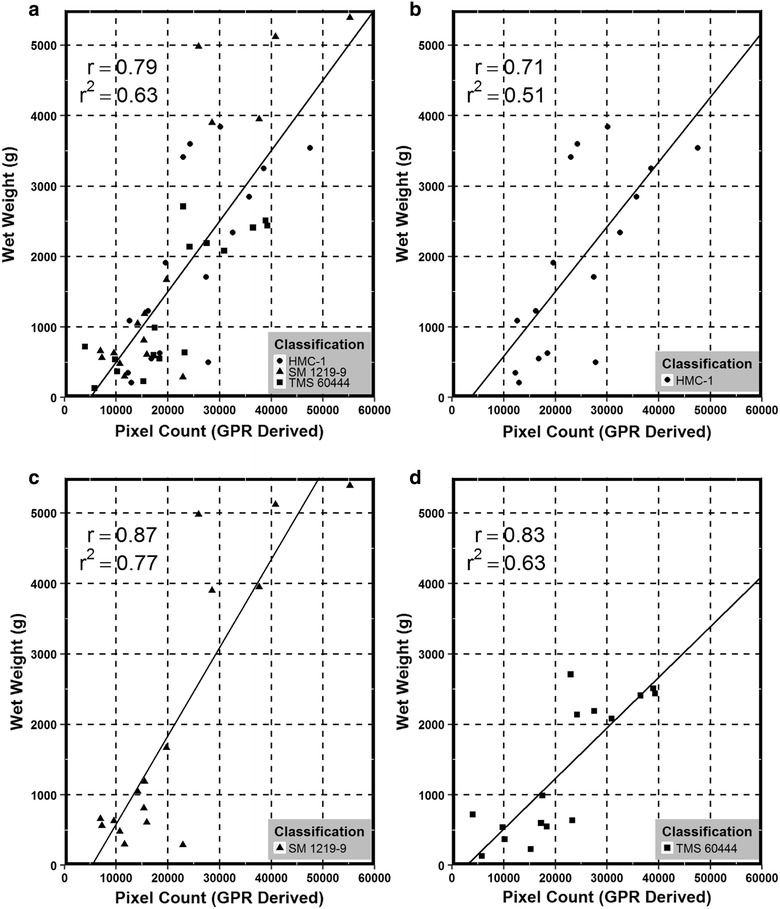
Linear regression of ground penetrating radar derived values for biomass (pixel count) and root fresh mass. Regression (**a**) is the general model in which all data samples were utilized, and regressions (**b**)–(**d**) are utilizing genotype-specific data (HMC-1, SM 1219-9, and TMS 60444 respectively). Regression equation, correlation coefficient, and coefficient of determination are provided for context

Figure 3 presents the linear regressions attained for each model where regression A is the general model in which all varieties were utilized for development, and regressions B–D are those for HMC, SM, and TMS respectively. The resulting correlation coefficient, the coefficient of determination, and significance level for each model can be found in Table 1. Table 1 provides convincing evidence that GPR is capable of estimating below ground biomass through a function of pixel counts. This is evident when looking at the high levels correlation coefficients and the significance of α < .001.

**Table 1:** The correlation coefficient (r), the coefficient of determination (r^2^), and significance level (*p*) for each linear regression model derived

Model Type	Genotype	r	r^2^	*p*
General	All Genotypes	.79	.63	<.001
Genotypic	HMC-1	.71	.51	<.001
Genotypic	SM 1219-9	.87	.77	<.001
Genotypic	TMS 60444	.83	.69	<.001

**Fig. 4:**
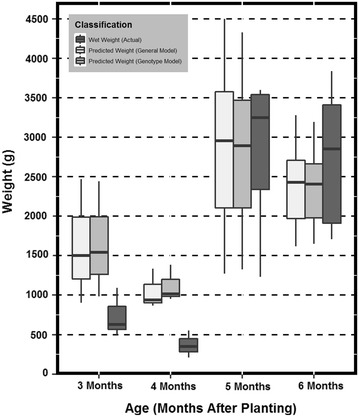
*Box* and *whisker plot* representations of the data distribution across age class for genotype HMC-1. The *box* and *whiskers* are filled based on the model type (general or genotypic) and the actual root fresh mass

**Fig. 5:**
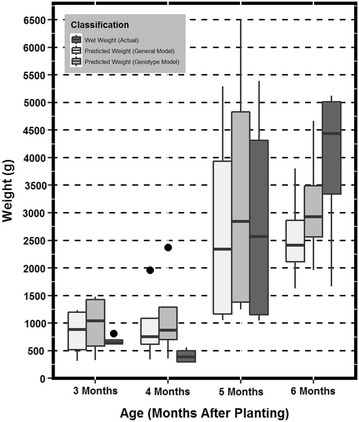
*Box* and *whisker plot* representations of the data distribution across age class for genotype SM 1219-9. The *box* and *whiskers* are filled based on the model type (general or genotypic) and the actual root fresh mass. Data outliers or any value greater than 1.5 times outside the interquartile range above or below are represented as *solid points*

**Fig. 6:**
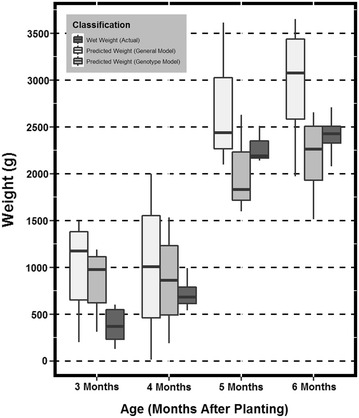
*Box* and *whisker plot* representations of the data distribution across age class for genotype TMS 60444. The *box* and *whiskers* are filled based on the model type (general or genotypic) and the actual root fresh mass

To test if GPR was capable of providing pertinent information specific to the root biomass a one-way analysis of variance test was performed. The variables compared were the RFM and the predicted RFM values from variety specific, and general models. The objective was to determine if the predicted RFM were found to be significantly different (α = .05) than the actual field weighed RFM. Figures 4, 5, and 6 can be utilized for visual interpretation of the results of the analysis of variance found in Table 2.

**Table 2:** Analysis of variance significance of F ratio results

Variety	Age (months)	Pr (> F)
HMC-1	3	.2253
	4	.0111*
	5	.9921
	6	.6897
SM 1219-9	3	.6268
	4	.3142
	5	.9227
	6	.3476
TMS 60444	3	.0772
	4	.8136
	5	.3765
	6	.1732

Investigating potential differences of root fresh mass (RFM) actual, general, and genotypic model predicted by variety and age

* Suggests significant difference (α = .05) between mass

Figures 4, 5, and 6 are box and whisker plot representations of the data distribution across age class for each of the three genotypes. The box and whiskers are filled based on the model type and the actual RFM for visual comparison. Data outliers or any value greater than 1.5 times outside the interquartile range above or below are represented as solid points as seen in Fig. 5. To create this data representation on a single scale, pixel data were converted to fit RFM using the newly derived regression equations. These equations can be found in Fig. 3. Results indicated that predicted mass (general and genotypic), were not significantly different than actual RFM (Table 2). Therefore, a general model approach can be utilized as a rapid method for measuring RBR.

To test if the measure derived utilizing the new general model could predict significant difference (α = .05) of RFM over time, a one-way analysis of variance was performed for the four time periods. This was also repeated utilizing the actual RFM to compare the results. Table 3 is a presentation of the resulting F ratio significance of an analysis of variance in which the actual and predicted RFM were tested for differences across time. The results infer that the general model was only sensitive enough when detecting differences in TMS, however, the other two cultivars were close to being significant considering that they were within ~.02% of probability from accepted value. The actual mass suggests no significant differences between months 3 and 4 or months 5 and 6. However, there were significant differences between months 3 and 4 against months 5 and 6.

**Table 3:** Analysis of variance significance of F ratio results

Variety	Type	*Pr* (>*F*)
HMC-1	Actual	.0016*
	Predicted	.0623
SM 1219-9	Actual	.0071*
	Predicted	.0732
TMS 60444	Actual	.0001*
	Predicted	.0026*

Investigating differing age groups for each genotype utilizing actual and general model predicted mass

* Suggests significant difference (α = .05) between age groups

## Discussions

The primary objective of the study was to determine if a function of GPR (pixel count) could be utilized to estimate RBR defined as the increase of RFM over time. The ability to detect root biomass non-destructively across different cassava varieties has a high potential to aid cassava breeders in the selection and release of new cultivars with rooting architectures that are favorable for planting (e.g. root area for increased planting density) and harvesting (e.g. steep rooting angles for reduced harvest damage) [32] as well as early bulking. It will also facilitate agronomic research to assess the impact of unique cultivation practices on root bulking. Detection of plants affected by diseases that affect root development such as Cassava Brown Streak Disease (CBSD) or Frog Skin Disease (FSD) could also be envisioned. These diseases (particularly FSD) do not induce symptoms in the above ground section of the plants, and thus infected plants cannot be roughed out and remain in the field serving as a source of inoculum) until harvest. These applications are only possible if the tool is sensitive enough to detect the subtle differences that occur in the growth of cassava roots. The discussed method has the potential to detect and measure root biomass with acceptable precision. Therefore, studies were also undertaken to test if the model was sensitive enough to capture the differences in RBR. These differences would detect when roots are growing and could then be associated with the environmental parameters to reduce the noise in the genotype by environment interaction. The general model was only able to detect this difference for TMS. Figures 4, 5, and 6 clearly suggest that the predictive models grossly overestimate RFM at months 3 and 4. This could be a potential cause for not detecting the differences between the early two ages versus the latter two. Also, when observing Figs. 4, 5, and 6 it can be inferred that TMS (Fig. 6) has smaller variance at every age as compared to HMC and SM (Figs. 4, 5 respectively). The level of sensitivity required for TMS is lower than that of the other two varieties and therefore it is easier to detect differences. One incident of significant difference between predicted and actual RFM was observed at one time period for one genotype only (Table 2). The genotype that exhibits this difference was HMC which was previously discussed to have discrepancies. These results would suggest that the general model would be reliable enough for identifying varieties with rapid root bulking rates.

### Root fresh mass detection

The objective of this study was to determine if there was correlation between RFM and pixel counts derived from a pixel thresholding procedure, and if so could one develop a linear regression model to utilize pixel counts to predict RFM. Having this ability, it would then be possible to predict RFM across time and therefore bulking rate of cassava. Since water has a greater dielectric constant than soil, the intensity of the amplitude response would be larger. Therefore, increased root moisture would facilitate the root detection process [33]. Also, not all varieties of cassava have the same root dry matter content [34]. So, utilizing dry mass would potentially reduce the correlation of GPR-derived variables and biomass. Based on these premises the concept of utilizing RFM versus dry mass was derived. This however creates a limitation to the utilization of GPR in field trials. The system currently does not have the ability to discriminate between moisture zones in the field and root moisture. Future study could consider a pre-planting scan for areas of greater soil moisture and develop a normalizing method to remove soil moisture noise from the post-planting field scans. It would also be necessary to consider the utilization of multi-array antennas for data acquisition that have variable frequencies to account for multiple root dimensions. The utilization of solely one frequency may cause the underestimation of roots that have a dimension less than the resolution of the frequency utilized. This would cause the regression analysis to become more sensitive to a specific age range. For this study however, the average bulked root diameter was greater than .03 m allowing for adequate detection with the utilized frequency. It should also be noted that the observed architecture of these varieties allowed for better acquisition and this may not be possible in all instances. Cassava varieties vary in architecture in which some may have a more downward vertical growth pattern that is not as visible to monostatic antennas such as the Detector Duo™. This however could be rectified in future study by utilizing a bistatic antenna that can detect across depth.

### Accounting for soil variability and anomalies

Previous studies have identified the potential for soil type and soil variability to create false positives in GPR data returns [35–38]. It is believed that due to the observed higher clay content and increased soil moisture in the plots scanned for HMC (field one), signal degradation occurred resulting in pixel misclassification. Also, all varieties were planted in non-sieved soils which were observed to have solid objects present to include rocks and clay clumps that could provide false positives in the data creating an overestimation [14]. Due to these observations, it was necessary to take a pre-processing step to remove outliers and prevent the incorporation of these false positives and poor data capture created by the field conditions. Data was tested for normality and the removal of outlier data was performed by removing all values that were two standard deviations away from the data mean. A future study should include a post-processing procedure to filter objects based on spectral characteristics rather than amplitude structure alone. This work is currently ongoing in other research fields and has had promising results [16, 39, 40].

### Model utilization

Regression analyses indicated that a significant positive correlation existed for the three varieties as a general model across the three clones. For one genotype (HMC), however, the model had a lower coefficient of determination (r^2^ = .51) than for the other two (SM and TMS). Conceptually a general model would be optimal for rapid capture of field data and minimal processing complexity by reducing the number of regression models needed for analysis. In the context of high-throughput phenotyping, more sensitive genotypic-based models or increased sample collection for calibration would create a lag in data capture and offset the potential time saved when utilizing a general model. Previous cassava modeling studies utilizing single or multiple above ground foliar parameters were found to be somewhat ineffective for measuring RBR [41]. It is often difficult to attribute above ground parameters (including phenological traits) to below ground functions due to the complex environmental interactions and the lack of knowledge on source-sink relationships in cassava [3, 5, 41]. By utilizing a tool that can capture information specific to the plant feature of interest (roots), it is possible to circumvent the problems encountered when utilizing indirect measurements such as leaf area index (LAI), plant height, or plant age.

## Conclusions

Previous studies have defined the capability of GPR to detect the positions of roots in both their horizontal and vertical positions [22, 42–48]. It had also established that GPR was capable of estimating root diameters and root dimensions [19, 46]. A foundation, therefore, had been established for further studies in root biomass estimation. Many different approaches for estimating root biomass had been established [27, 49–54], but due to the empirical nature of these studies a new dataset was necessary to conclusively determine the tools capability in cassava root biomass estimation for establishing RBR. We specifically sought to define a particular model that would facilitate monitoring growth of cassava biomass, RBR, for the selection of early bulking genotypes. In conclusion, it was possible to determine with precision at what time period cassava was increasing root biomass. This allows plant breeders and agronomists to non-destructively sample root biomass by attaining GPR returns and applying the regression equation derived from the empirical model rather than through destructive harvesting of roots. Cassava, often harvested between 10 and 12 months after planting, generally follows a sinusoidal growth pattern for root bulking and therefore will have drastic changes in mass over time [41]. The model presented does not account for the complete growth cycle since late onset bulking, when changes in RFM may be more drastic (e.g. from 8 to 12 months) [55], was not considered for this early bulking study. In future studies, data should be collected across all time periods and a sinusoidal model should be developed to better fit the plant as a whole and potentially be utilized to determine a bulking threshold, or at what point did bulking rate plateau. Though results presented defined the potential for GPR to calculate root bulking characteristics, additional work is required. Issues with overall mass correlations and improved soil filtering methodologies are needed to reduce secondary soil anomalies that can cause inaccurate pixel classification. Measurements of exact root position (at depth) were not possible due to time constraints in the field, therefore, no accuracy assessment was possible for the image sub-sampled threshold values. Also, some limitations in tool functionality do exist. Some limitations include the physical properties of the signal and the media in which the signal travels. GPR signal is hindered by the sheeting structure of clays that cause the frequency to be dissipated as heat and reduces the available energy returned to the receiving antenna [10]. Therefore, any root architecture parallel to the signal polarity may create refractions and full returns are lost, causing inaccurate estimations of root mass [10]. Future studies should consider these flaws and would benefit from the incorporation of ancillary data such as soil pre-planting analysis and above ground phenology, advanced data pre-processing to reduce the error encountered by utilization of amplitude response alone, and newer antenna designs that do not require ground coupling and destructive sampling.

## Abbreviations

G × E: genotype by environment interaction
GPR: ground penetrating radar
RBR: root-bulking rate
HT: high throughput
CIAT: International Center for Tropical Agriculture
m.a.s.l: meters above sea level
HH Polarization: horizontal transmit and horizontal receive polarization
HMC: variety HMC-1
SM: variety SM 1219-9
TMS: variety TMS-60444
RFM: root fresh mass
LAI: leaf area index
